# Laboratory routine in the ICU: a practice to be abolished?

**DOI:** 10.1186/cc12628

**Published:** 2013-06-19

**Authors:** AA Peixoto, FA Meneses, BP Barbosa, LFP Pessoa, RHT Melo, GM Fideles

**Affiliations:** 1 Centro Universitário Christus, Papicu, Fortaleza, CE, Brazil; 2 Hospital Universitário Walter Cantídio - UFC, Papicu, Fortaleza, CE, Brazil; 3 Hospital Geral de Fortaleza - SESA, Papicu, Fortaleza, CE, Brazil

## Introduction

The execution of laboratory tests is frequently requested for diagnosis and/or monitoring of critical patients. Their role as an aid, however, is diminished by unreasonable practices - resulting, unfortunately, in iatrogenesis and higher costs. In face of these ominous results, we tracked the laboratory tests performed routinely in an ICU.

## Methods

We retrospectively analyzed the results of tests performed on a daily and consecutive basis during the months of May and June 2012. We deliberately restricted our analysis to the levels of creatinine, urea, sodium, and potassium, and to the prothrombin time (PT) and activated partial thromboplastin time (aPTT) tests. To infer their propriety, we compared each result with their respective normality references. In addition to comparing the cumulative rates of normal tests, we were also interested in the correlation of the results and the admission APACHE II score and time of hospitalization in the ICU, as well as in the volumes of blood drawn and in the test costs.

## Results

Forty-eight patients (28 men) were studied, with a mean age of 46.6 ± 18.6 years, average APACHE II score of 16.5 ± 6.9 points, average length of stay in the ICU of 15.2 ± 11.7 days, and 33% fatality rate. A total of 3,622 tests were performed (90.6/deceased patient × 67.1/ surviving patient), with a predominance of potassium (13.5%), sodium (13.3%), and creatinine (13.3%) levels, and complete blood count, (13.2%). We observed a linear correlation (*r *= 0.81; *P <*0.05) between the number of performed tests/patient and the respective length of stay in the ICU; no correlation was observed for the APACHE II score. The volume of blood drawn per patient per hospitalization varied between 10 and 525 ml, being higher for deceased patients (average of 103.5 × 84.2). Of the total tests, 48.8% of the results were normal (43.9 tests/surviving patient × 44.9 tests/deceased patient), especially for potassium levels (9.8%) and aPTT (9.2%). Also, 31.8% of all results were consecutively normal, specially potassium levels (7.1%) and aPTT (6.8%). A total of approximately US$65,000 was spent in tests for which results were normal.

## Conclusion

The collected data reveal that almost one-half of the tests resulted in normal values, with the aggravating factor of consecutivity. This authorizes us to question the routine practice of laboratory tests, highlighting their possible meaninglessness for decision-making, as well as their implication in unreasonable blood spoliation (culminating in transfusions) and their respective costs (approximately US$4,000 annually).

